# Comprehensive Pulmonary Rehabilitation for Patients with Malignant Pleural Mesothelioma: A Feasibility Pilot Study

**DOI:** 10.3390/cancers16112023

**Published:** 2024-05-26

**Authors:** Lorenzo Lippi, Alessandro de Sire, Arianna Folli, Claudio Curci, Dario Calafiore, Mariano Lombardi, Luca Bertolaccini, Alessio Turco, Antonio Ammendolia, Nicola Fusco, Lorenzo Spaggiari, Marco Invernizzi

**Affiliations:** 1 Department of Scientific Research, Campus LUdeS Lugano (CH), Off-Campus Semmelweis University of Budapest, 1085 Budapest, Hungary; lorenzo.lippi@uniludes.ch; 2 Department of Medical and Surgical Sciences, University of Catanzaro “Magna Graecia”, 88100 Catanzaro, Italy; ammendolia@unicz.it; 3 Research Center on Musculoskeletal Health, MusculoSkeletalHealth@UMG, University of Catanzaro “Magna Graecia”, 88100 Catanzaro, Italy; 4 Department of Health Sciences, University of Eastern Piedmont “A. Avogadro”, 28100 Novara, Italy; arianna.folli@ospedale.al.it (A.F.); alessio.turco@ospedale.al.it (A.T.); marco.invernizzi@med.uniupo.it (M.I.); 5 Physical Medicine and Rehabilitation Unit, Department of Neurosciences, ASST Carlo Poma, 46100 Mantova, Italy; claudio.curci@asst-mantova.it (C.C.); dario.calafiore@asst-mantova.it (D.C.); 6 Division of Pathology, IEO European Institute of Oncology IRCCS, 20139 Milan, Italy; mariano.lombardi@ieo.it (M.L.); nicola.fusco@unimi.it (N.F.); lorenzo.spaggiari@unimi.it (L.S.); 7 Division of Thoracic Surgery, IEO European Institute of Oncology IRCCS, 20139 Milan, Italy; luca.bertolaccini@gmail.com; 8 Department of Oncology and Hematology-Oncology, University of Milan, 20122 Milan, Italy; 9 Translational Medicine, Dipartimento Attività Integrate Ricerca e Innovazione (DAIRI), Azienda Ospedaliera SS. Antonio e Biagio e Cesare Arrigo, 15121 Alessandria, Italy

**Keywords:** malignant pleural mesothelioma, physical function, muscle, complementary treatment, physical exercise, rehabilitation

## Abstract

**Simple Summary:**

Rehabilitation might play a crucial role in malignant pleural mesothelioma (MPM), but its role is still debated in MPM patients. The intervention comprised multidisciplinary educational sessions, physical rehabilitation, and respiratory physiotherapy. Feasibility was evaluated based on dropout rates, adherence to the rehabilitation program, safety, and patient-reported outcomes. In light of the current evidence, we have conducted a pilot study to assess the feasibility of tailored pulmonary rehabilitation in terms of physical and respiratory function in MPM. Twelve patients were initially enrolled, with seven completing the study. High adherence to physical (T1: 93.43%, T2: 82.56%) and respiratory (T1: 96.2%, T2: 92.5%) rehabilitation was observed, with minimal adverse events reported. Patient satisfaction remained high throughout the study (GPE scores at T1: 1.83 ± 1.17; T2: 2.0 ± 1.15), with improvements noted in physical function, pain management, and health-related quality of life. Despite its promising outcomes, further research with larger samples is warranted to validate its efficacy and integrate rehabilitation as a component into the multidisciplinary management of MPM.

**Abstract:**

Malignant pleural mesothelioma (MPM) represents a significant health burden, with limited treatment options and poor prognosis. Despite advances in pharmacological and surgical interventions, the role of rehabilitation in MPM management remains underexplored. This study aims to assess the feasibility of a tailored pulmonary rehabilitation intervention addressing physical and respiratory function in MPM patients. A prospective pilot study was conducted on surgically treated MPM patients referred to a cardiopulmonary rehabilitation service. The intervention comprised multidisciplinary educational sessions, physical rehabilitation, and respiratory physiotherapy. Feasibility was evaluated based on dropout rates, adherence to the rehabilitation program, safety, and patient-reported outcomes. Twelve patients were initially enrolled, with seven completing the study. High adherence to physical (T1: 93.43%, T2: 82.56%) and respiratory (T1: 96.2%, T2: 92.5%) rehabilitation was observed, with minimal adverse events reported. Patient satisfaction remained high throughout the study (GPE scores at T1: 1.83 ± 1.17; T2: 2.0 ± 1.15), with improvements noted in physical function, pain management, and health-related quality of life. However, some issues, such as time constraints and lack of continuous supervision, were reported by participants. This pilot study demonstrates the feasibility and potential benefits of a tailored pulmonary rehabilitation intervention in MPM patients. Despite its promising outcomes, further research with larger samples is warranted to validate its efficacy and integrate rehabilitation as a component into the multidisciplinary management of MPM.

## 1. Introduction

Malignant pleural mesothelioma (MPM) is a rare cancer occurring in 5–6/100,000 patients per year and is characterized by a poor survival ranging between 13.3 and 20.2 months [1,2,3]. In recent years, MPM incidence has been rising due to the extensive use of asbestos, which represents the main risk factor for MPM development [2,4]. To date, the combination of multi-targeted antifolate and platinum compound is considered the first-line intervention in the pharmacological treatment of the disease, and several studies support the positive effects in overall survival [5,6,7,8,9,10,11,12]. On the other hand, immunotherapy has recently been proposed for the management of MPM. However, little evidence supports its benefits, and further investigations are needed to assess its role [13]. In this context, thoracic surgery plays a key role in improving the symptom management of patients with MPM. Recent guidelines support its positive effects in a comprehensive approach to MPM management [14]. 

Despite surgical and pharmacological approaches being deeply studied options in the management of patients with MPM, there is an increasing interest in the rehabilitation field to improve MPM symptom management and the health-related quality of life (HR-QoL) of patients with MPM. 

In particular, dyspnea is one of the most common symptoms noted by these patients, which is frequently associated with malignant pleural effusion [15]. Moreover, cancer pain might be often related to the direct invasion of the pleura or the chest wall [16]. Lastly, functional impairment and decreased physical performance are strictly associated with MPM surgery, with a significant impact on HR-QoL [17]. Despite these considerations, the physical and psychosocial consequences of MPM are underestimated and poorly addressed issues, probably due to the rareness of the disease and the poor prognosis [17]. Moreover, the gap in knowledge about specific pulmonary rehabilitative interventions combined with the lack of dedicated clinical pathways has severely influenced the rehabilitation approach in this condition [18,19].

However, taking into account the detrimental clinical, emotional, and social burden of MPM, the prompt and effective management of its disabling sequelae is mandatory to improve HR-QoL and the health status of both MPM patients and caregivers. In this scenario, physical activity and rehabilitation interventions have been widely proposed as effective non-pharmacological therapies in the complex management of cancer-related functional and disabling sequelae [20,21]. However, there still needs to be more consensus about the precise and specific rehabilitative exercise protocols to be performed, as there are no data about specific rehabilitation interventions in the long-term management of MPM patients.

Therefore, primary aim of this pilot study was to assess the feasibility of a tailored pulmonary rehabilitation intervention in maintaining or improving physical and respiratory function in individuals with MPM. Specifically, this study aimed to (1) evaluate the adherence to and acceptability of the intervention, (2) measure changes in physical function, and (3) measure changes in respiratory function. We hypothesized that participants undergoing the tailored pulmonary rehabilitation intervention would show improvements in both physical and respiratory function, providing a basis for clinically relevant recommendations for evidence-based rehabilitation in this disabling condition.

## 2. Materials and Methods

### 2.1. Study Design and Participants

In this prospective pilot study, we assessed a consecutive series of patients surgically treated for MPM and referred to the Cardiopulmonary Rehabilitation Service of the Azienda Ospedaliera “SS. Antonio e Biagio e Cesare Arrigo” of Alessandria, Italy, between March 2022 and February 2023. 

The study protocol was developed following the SPIRIT guidelines [22]. Inclusion criteria were: (a) age ≥ 18 years old; (b) signed informed consent; (c) diagnosis of MPM; (d) previous talc-pleurodesis or pleurectomy/decortication; (e) expected survival over 6 months; and (f) Mini-Mental State Examination [23] score ≥ 24. Exclusion criteria were as follows: (a) Karnofsky performance status [24] below 60%; (b) absolute contraindications for physical activity; (c) brain metastases; (d) bone metastases compromising the stability of skeletal segments involved in the treatment; (e) pregnancy or breastfeeding; and (f) failure to sign the informed consent form.

The eligibility was assessed by an expert physician specialized in physical and rehabilitation medicine. Approval of the trial protocol (ASO.RiabCR.21.02; Protocol code: SAFE-MESO) was obtained from the Institutional Review Board and was performed in accordance with the Declaration of Helsinki [25] and pertinent national and international regulatory requirements. All the participants were asked to carefully read and sign an informed consent form and were allowed to ask questions about the study.

The manuscript was written according to the STROBE (strengthening the reporting of observational studies in epidemiology) statement [26].

### 2.2. Intervention

**Figure 1:**
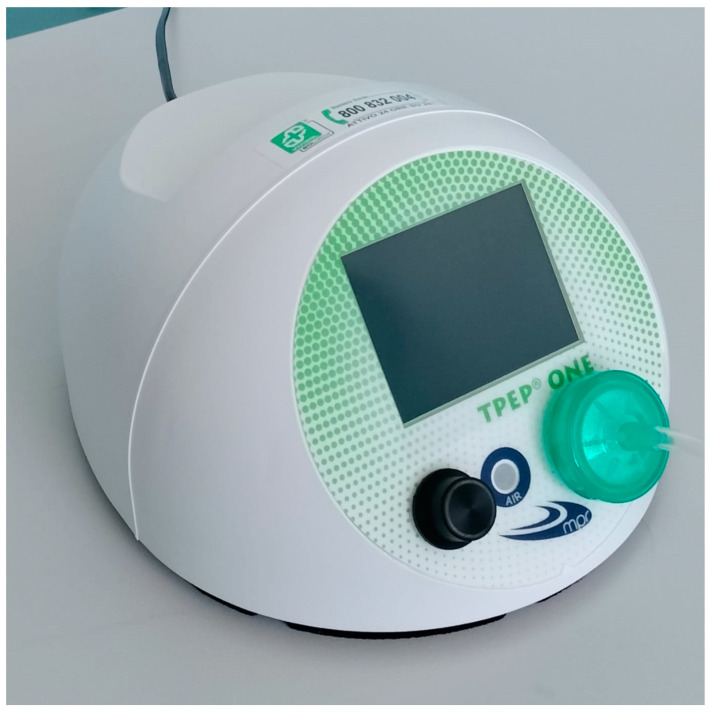
TPEP^®^ ON.

After baseline assessments, participants meeting the eligibility criteria underwent a multi-target rehabilitation protocol composed of the following elements:

- Counseling and educational therapy. All patients included underwent a single multidisciplinary educational session conducted by different healthcare operators (physiatrist, physiotherapist, nurse, speech therapist, and dietician), focusing on energy conservation strategies in activities of daily living (ADL), symptom recognition and management, and training for respiratory devices (i.e., oxygen therapy, aerosol therapy, etc.).
- Physical rehabilitation. Patients were subjected to a home-based physical rehabilitation protocol consisting of 50–60 min sessions three times a week. The sessions were structured as follows: (1) warm-up phase (5–10 min), consisting of stretching and active joint mobilization exercises; (2) resistance-exercises phase with body weight and a TheraBand, targeting all major muscle groups at 60–75% and an estimated one-repetition maximum (1RM) for approximately 20 min; (3) aerobic-exercise phase targeting an exercise intensity between 60 and 85% of maximal heart rate, based on the patient’s tolerance. Activities included walking, swimming, and cycling for at least 20 min; and (4) cool-down phase (5–10 min) with stretching and active joint mobilization exercises. The first three sessions were supervised by an experienced physical therapist, who showed the correct execution of physical exercises. A booklet including pictures and instructions on the physical rehabilitation program was provided to support patients in the home-based rehabilitation program, and progress was recorded in a self-treatment diary to monitor adherence to the rehabilitation program.
- Respiratory physiotherapy. The respiratory physiotherapy program included respiratory muscle training, lung recruitment maneuvers, and bronchial secretion management using a specific device (Temporary Positive Expiratory Pressure—TPEP^®^ ONE, Legnano, Milan, Italy) twice daily for 15 min (as depicted in Figure 1).

The device is a portable technology delivering low positive expiratory pressure in the mouth during spontaneous breathing. The Inspiration/Expiration (I/E) mode [27] allows for deep lung recruitment with a positive expiratory pressure through real-time visual feedback displayed on a screen, which shows the flow-dependent expiratory and/or inspiratory resistances. The patients were instructed to perform cycles of deep, slow inspiration followed by a slow expiration. Moreover, the device has a “time adaptation” function, which calculates the average time of the I/E cycle and stimulates the patient to improve his performance based on the I/E time. The main objective of each cycle was to achieve and maintain the I/E goals for as long as possible, ideally by progressively increasing the duration of the cycles. If the patient needed a pause or could not maintain performance, they were advised to stop exercising for 1–2 min. In addition to TPEP^®^ ONE rehabilitation training, educational therapy focusing on postural and breathing strategies was administered to each study participant to minimize physical stress during ADLs; in addition, targeted postural exercises, stretching, and relaxation techniques were shown. 

Patient progressions were assessed during follow-up assessments (T1 and T2), while the multi-target rehabilitation program was tailored to the patient’s improvements and disease progression.

The rehabilitation treatment was suspended when the patient no longer met inclusion or exclusion criteria due to the clinical worsening of the disease or evolution in general clinical conditions.

### 2.3. Quantitative Outcome Measures

Sociodemographic and anthropometric data were collected at baseline (T0). Primary and secondary outcomes were assessed at baseline, 1 month (T1), and 6 months (T2).

According to the pilot design, the primary outcome was the feasibility of the multi-target rehabilitation program, assessed by the number of dropouts; adherence to the rehabilitation program; and the safety of the study intervention. More specifically, the number of dropouts was registered at any point in time. Patients missing any follow-up assessment were interviewed by phone to explain their dropout. A specific adherence diary recording home-based rehabilitation sessions assessed rehabilitation program compliance. Patients undergoing less than 80% of training sessions were registered as dropouts. The safety of the study intervention was evaluated by self-reported adverse events. Lastly, the Global Perceived Effect (GPE) scale was used to characterize patients’ perceived effects of the study intervention at T1 and T2; it consists of a seven-item Likert scale with scores ranging from 1 (best satisfaction) to 7 (unsatisfaction).

The secondary outcome measures were as follows:

- Physical functioning: lower-limb physical performance was assessed by the Short Physical Performance Battery (SPPB) [28]; submaximal exercise capacity, measured through a 30 s sit-to-stand (STS) test [29]; a two-minute-walking test (2MWT) [30,31] for the detection of dyspnea (Borg CR10 [32]); and oxygen saturation (SpO_2_), and muscle strength was assessed by a handgrip strength test (HST) using Jamar^®^ dynamometer [33,34].
- Pulmonary function: assessed by arterial blood gas (ABG) analysis (characterizing partial pressure of oxygen [PaO_2_], partial pressure of carbon dioxide [PaCO_2_], and pH) and spirometry, which assessed forced expiratory volume in the first second (FEV1), forced vital capacity (FVC), diffusing capacity for carbon monoxide (DLCO), and peak expiratory flow (PEF).
- Pain intensity: pain was assessed through the Visual Analog Scale (VAS) [35].
- Nutritional assessment: nutritional screening was conducted through the Mini Nutritional Assessment (MNA) [36].
- Health-related quality of life (HR-QoL): evaluated by the European Organization for Research and Treatment of Cancer Quality of Life Questionnaire (EORTC QLQ–C30), a scale composed of a 30-item questionnaire, including a functional scale (physical, role, cognitive, emotional, and social), a symptom scale (fatigue, pain, and nausea and vomiting), and a global QoL scale. Single items assessed further symptoms frequently reported by oncologic patients (such as dyspnea, loss of appetite, insomnia, constipation, and diarrhea) and the perceived financial impact of the disease; there were four possible answers: “Not at all”, “A little”, “Quite a bit” and “Very much” [37]. To better characterize QoL in patients with MPM, the Lung Cancer Symptom Scale-Mesothelioma (LCSS-meso) was used in included patients [38]. This nine-item site-specific QoL measure concentrates on six symptoms (appetite loss, fatigue, cough, dyspnea, hemoptysis, and pain) and three summary items (symptomatic distress, normal activity, and global QoL). All items are measured using 100 mm lines to assess the intensity of patient responses (with 0 as the lowest and 100 mm as the greatest value) in the previous 24 h. The total score is obtained by the average of all nine item scores [38]. Patient perspective on physical, mental, and social well–being was assessed through the Patient-Reported Outcomes Measurement Information System (PROMIS) based on questions in a 7-day recall period, exploring items such as anxiety, anger, depression, fatigue, pain quality, pain interference, pain behavior, satisfaction with participation in discretionary social activities, satisfaction with the involvement in social roles, sleep disturbance, and sleep-related impairment, with five response options (e.g., 1 = Not at all, 2 = A little bit, 3 = Somewhat, 4 = Quite a bit, 5 = Very much) [39].

### 2.4. Qualitative Analysis

All participants were asked to answer a qualitative questionnaire composed of 23 different items to provide qualitative data about the proposed rehabilitation intervention.

The qualitative questionnaire was realized by an expert consensus of 10 different operators with years of experience in the management of patients with MPM. The qualitative questionnaire was performed with an inductive approach to characterize the content of the data through open coding, creating categories, and abstracting to main categories according to previous studies [40,41].

The qualitative questionnaire was composed of four domains: (A) perceived effectiveness of the rehabilitation intervention, characterizing benefits in symptom management during the study; (B) barriers that mainly affected the adherence to rehabilitation treatment; (C) rehabilitation impact on quality of life, characterizing time spent in rehabilitation treatment and the effects of physical and psychosocial wellbeing; and (D) psychological and social experiences related to the treatment, characterizing the potential improvements and personalization of the study rehabilitation intervention.

Data analysis was performed by an operator without previous interaction with patients. More information about the qualitative questionnaire is shown in Supplementary Material S1.

### 2.5. Statistical Analysis

Statistical analysis was performed using GraphPad Prism version 7.00 (GraphPad Software, La Jolla, CA, USA). As a pilot feasibility study, the sample size calculation was optional. According to a previous study [42], a non-Gaussian distribution was assumed due to the low numerosity of the sample. Categorical variables were expressed as numbers and ratios, while continuous variables were expressed as means ± standard deviations. Wilcoxon’s signed-rank test assessed the differences in quantitative outcome measures between different time points. Minimum clinically important difference (MCID) was used to characterize clinical implications related to significant changes in outcome measures. A type I error level of 0.05 was chosen. A qualitative description of the results of the questionnaire administered at T2 was included. A *p*-value lower than 0.05 was considered statistically significant.

## 3. Results

**Figure 2:**
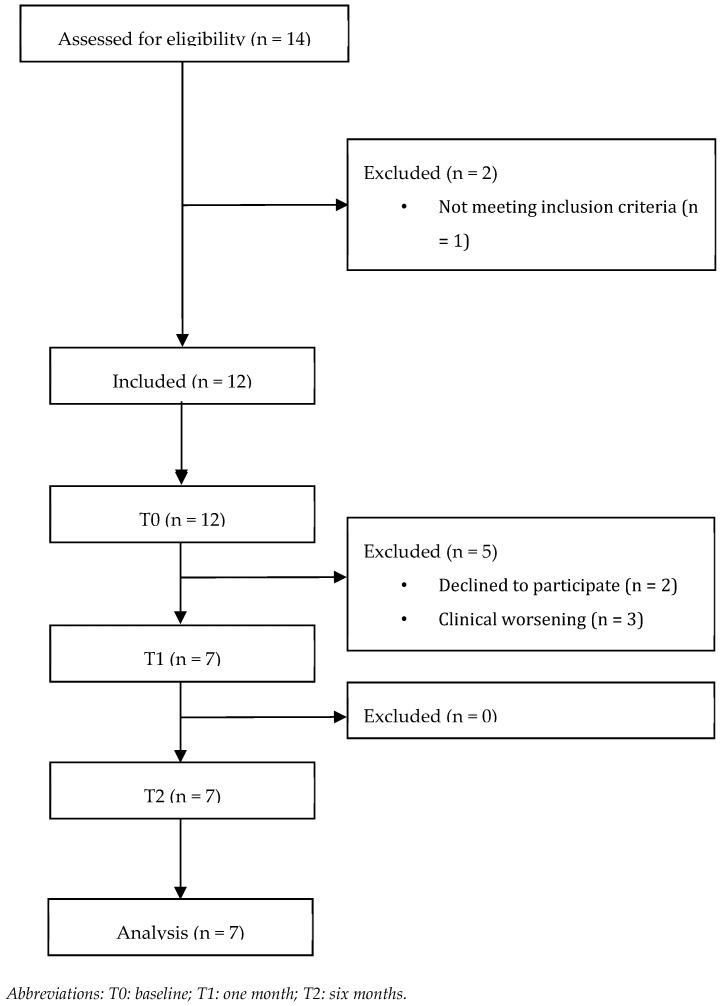
Patient flow diagram.

Out of 14 patients assessed for eligibility, 12 fulfilled the inclusion criteria and were evaluated at T0. Five patients were lost at T1 for refusal of further participation (*n* = 2) and worsening of clinical conditions (*n* = 3). As a result, seven patients (*n* = 7) completed the study and were included in the analysis (Figure 2 shows the study flowchart).

The study population comprised a homogeneous sample of patients with MPM: six males and one female with a mean age of 67 ± 6 years and a mean BMI of 26.05 ± 5.04 kg/m^2^. Table 1 reports further details about the characteristics of the patients included.

### 3.1. Primary Outcomes

Adherence to the physical rehabilitation program was high overall, with a mean adherence rate of 93.43% at T1 and 82.56% at T2. On the other hand, a higher adherence rate was reported for the respiratory physiotherapy program, with 96.2% of sessions completed at T1 and 92.5% at T2. No major adverse events were reported. Few minor side effects were reported, with a mean of side effects of 0.57 ± 0.53 at T1 and 0.71 ± 0.76 at T2. The minor side effects reported were coughing (T1: *n* = 2; T2: *n* = 4), dizziness (T1 *n* = 1), and transient muscular pain (T1: *n* = 1; T2: *n* = 1). 

The GPE score at T1 was 1.83 ± 1.17, characterizing high satisfaction. On the other hand, a low decrease in patient satisfaction was reported at T2 (GPE score: 2 ± 1.15).

### 3.2. Secondary Outcomes

#### 3.2.1. Physical Functioning

The main results for physical performance parameters are reported in detail in Table 2. No significant changes were reported in terms of HST and SPPB at all time points. In contrast, there was a significant improvement in the 30 s STS test (*p* = 0.047) at T2, which showed a higher increase than the MCID [43]. On the other hand, a significant worsening of SpO2 during 2MWT was reported (*p* = 0.031). No significant differences were reported in 2MWT measures, albeit a consistent improvement in terms of 2MWT distance was reported at T1 (17.43 ± 23.86 m), even higher than MCID [44].

#### 3.2.2. Blood Gases and Pulmonary Function

Blood gases and pulmonary function data are summarized in detail in Table 3. The arterial gas analysis did not show significant changes. Concurrently, pulmonary function parameters did not show significant changes. On the other hand, it should be noted that no worsening trend was highlighted in either arterial gas analysis or pulmonary function.

#### 3.2.3. Multidimensional Assessment of Patients with MPM

The results of the multidimensional assessment of patients with MPM are summarized in Table 4.

Pain intensity showed a reduction after the study intervention despite the fact it did not reach statistical significance. However, the decrease in pain intensity (T0-T1: 11.86 ± 18.33 and T0–T2: 10.71 ± 22.07) was higher than the MCDI of 9.9 [37]. The MNA showed an appropriate nutritional status was maintained until the end of the study, with no significant changes between different time points.

LCSS-meso showed a significant worsening at T2 when compared to T0 (T0–T2: −7.70 ± 7.86, *p* = 0.047). On the other hand, EORTC QLQ-C30 and PROMIS had a non-significant tendency toward improvement, without significant differences between time points. However, the PROMIS scale showed an improvement higher than the MCID [45] at both time points (T0–T1: 10.14 ± 31.20, T0–T2: 15. 29 ± 26.54).

### 3.3. Qualitative Analysis

The qualitative analysis was divided into four main domains: perceived effectiveness, barriers to rehabilitation, impact on quality of life, and psychological and social experiences.

#### 3.3.1. Perceived Effectiveness

Patients were relatively consistent across all fields explored, meaning that those who found it helpful had a more positive attitude. In contrast, the ones who found it unhelpful had a more negative mindset.

“*Since I started the rehabilitation program, I have had improvements in several settings. I still can do the things I did during the first assessments. […] Two weeks ago, I went for a CT scan. Unfortunately, there was no improvement […], but this is not the fault of the therapy. I enjoyed therapy and enjoyed doing it*”.

(*Patient ID 3*)

*“I haven’t felt any benefits from rehabilitation therapy, even now I’m doing it. However, I have no perception that it is of any use”.*

(*Patient ID 5*)

#### 3.3.2. Barriers to Rehabilitation

Fitting a new routine into daily life appeared as the most critical barrier, often appearing as the most negligible task in their new life with cancer. More in detail, the time needed to learn and execute the exercises was the most critical issue.

*“The rehabilitation program was time spending. I still worked several hours a day […] it was a challenge to find time to fit everything together”. *

(*Patient ID 5*)

Moreover, there needed to be more continuous supervision.

*“The program was difficult at the beginning. […] I need a person who follows me and teaches me well. It is important for the elderly when they start to lack memory and desire to do things.”*

On the other hand, no concerns emerged about the utilization of TPEP^®^.

*“The rehabilitation program had no interactions with family. […] We went away for a couple of days; I took the device with me, and I did rehabilitation in the hotel”.*

(*Patient ID 6*)

#### 3.3.3. Impact on Quality of Life

Overall, the majority of patients did not report a negative impact on their quality of life, although no great benefit was reported.

*“The program had no impact on my free time; I did it when I was free”. *

(*Patient ID 1*)

One patient complained that his social life had significantly decreased.

*”My social life has reduced a lot, probably also because of the COVID pandemic […] Rehabilitation treatment itself takes up a lot of time”. *

(*Patient ID 1*)

#### 3.3.4. Psychological and Social Experiences

Some patients reported positive effects in symptom control, with positive implications in anxiety and distress.

*“The rehabilitation program was fine, it was simple. […] When I finished the program, I breathed better, and I felt more calm”. *

(*Patient ID 6*)

A significant feature was the inability to differentiate between a lack of improvement due to the treatment and the worsening of the underlying disease. On the other hand, patients had expectations of the treatment. When the feedback relating to the promised care did not live up to those expectations, it could have created frustration and a negative attitude.

## 4. Discussion

In this pilot study, seven patients completed the study. Adherence rates were high for both physical (T1: 93.43%, T2: 82.56%) and respiratory (T1: 96.2%, T2: 92.5%) rehabilitation programs, with minimal side effects reported. Patient satisfaction, as measured by GPE scores, was high at T1 (1.83 ± 1.17) and slightly decreased at T2 (2 ± 1.15). Significant improvement in the 30 s STS test was observed at T2 (*p* = 0.047), while SpO2 levels during 2MWT worsened (*p* = 0.031). No significant changes were noted in 2MWT distance or pulmonary function parameters. Pain intensity decreased, though not significantly. LCSS-meso showed a significant worsening at T2 (*p* = 0.047), while PROMIS improvements exceeded MCID at both time points. Qualitative analysis revealed positive effects on symptom control but noted barriers to rehabilitation integration into daily life. Patients reported positive experiences with the intervention overall, but some challenges remain, particularly regarding routine integration and time constraints.

MPM is a rare tumor linked to asbestos exposure with a peculiar incidence pattern (geographical clusterization). To date, MPM is considered an unmet need due to the lack of effective therapeutic options and poor overall survival [46]. In this context, physical function impairment might further worsen outcomes, with recent studies suggesting that physical performance assessment and rehabilitation should be integrated into the routine clinical practice of patients with MPM [47]. In accordance, growing research is currently highlighting the crucial role of interdisciplinary and transdisciplinary rehabilitation interventions in addressing cancer-related symptoms, also using novel technological approaches [48,49]. However, despite these considerations, there is still a gap in our knowledge of the optimal rehabilitative management of cancer patients, while studies assessing the effects of a comprehensive rehabilitation intervention in a homogeneous sample of patients with MPM are still lacking. Thus, this study aimed to determine the feasibility of a comprehensive rehabilitation intervention to provide clinically relevant evidence about the complementary rehabilitation management of people with MPM.

Interestingly, our data showed promising results in terms of physical functioning assessed by a 30 s STS and 2MWT. These findings are significant given the recent systematic review and meta-analysis by Nakano et al. [50], which underlined the strict relation between physical function and mortality in cancer patients. Moreover, it has been reported that patients suffering from thoracic cancer might be less physically active than healthy individuals, with detrimental consequences in both physical and pulmonary function [51]. Our findings showed no significant changes in terms of ABG analysis and spirometry. In this context, MPM is a progressive disease characterized by local expansion commonly associated with decreased FEV1 and FVC [52,53,54].

On the other hand, there are no data about the optimal rehabilitation intervention reducing the breathiness and dyspnea of people with MPM, and currently available data on pulmonary rehabilitation interventions concern heterogeneous samples composed of patients with different thoracic cancers [55,56,57,58,59]. Interestingly, our cancer-specific data suggest that a comprehensive rehabilitation intervention might be considered a feasible option in the management of physical and pulmonary function impairment. However, further studies assessing larger samples are needed to elucidate the role of specific respiratory physiotherapy interventions in improving physical and pulmonary function in people with MPM.

In addition, a positive trend was reported in pain management in terms of VAS scores. In this scenario, pain is one of the most common symptoms complained about by patients suffering from MPM, crucially affecting HR-QoL [14]. Due to its multifactorial etiology, pain management in MPM patients is still challenging. Our data suggest that a complementary rehabilitation intervention might be considered as an add-on to conventional therapy to reduce pain intensity in people with MPM. 

Patients suffering from thoracic cancer might be characterized by reduced muscle strength and nutritional status impairment, leading to increased disability and crucially affecting HR-QoL [51]. More in detail, nutritional status and HR-QoL were assessed in the recent study by Jeffery et al. [60], in which a homogeneous sample of 61 patients suffering from MPM was prospectively evaluated. The authors reported that patients with a lower HR-QoL had a higher risk of malnutrition (*p* < 0.001).

Interestingly, our data suggest a non-significant tendency toward improvement in terms of HR-QoL, with an MCID reached by the PROMIS scale. These data align with previous studies supporting the positive effects of a comprehensive rehabilitation strategy in improving the HR-QoL of patients with cancers [19]. 

Previous studies assessed the cancer-specific effects of rehabilitation in patients with MPM. In particular, the recent studies by Tanaka et al. [61,62] evaluated the impact of an early rehabilitation approach after thoracic surgery in patients with MPM. However, the authors considered a small sample only a few days after surgery with in-patient rehabilitation. To the best of our knowledge, no previous study provided data about the long-term effects of a home-based rehabilitation program for people with MPM. Moreover, a qualitative analysis of a comprehensive rehabilitation intervention of a homogeneous sample of patients with MPM was still lacking. 

In the scientific literature, there is a growing interest in the role that pulmonary rehabilitation might play, particularly in chronic obstructive pulmonary disease, a burdensome condition affecting several people worldwide [63,64,65,66]; however, we showed how the reduction of functional impairment and HR-QoL should be solved by a complementary management of patients with thoracic cancer. However, the effects of rehabilitation on MPM are still debated, and no previous study has assessed the long-term effects of a home-based rehabilitation program in terms of physical functioning and HR-QoL.

In summary, the observed improvements in physical functioning, symptom management, and quality of life suggest the potential benefits of comprehensive rehabilitation interventions in enhancing the overall well-being of MPM patients.

Our study employed a single-arm pilot design to assess the feasibility and preliminary effectiveness of a tailored pulmonary rehabilitation intervention for individuals with MPM. The adherence rate of the interventions suggests potential replicability in similar clinical settings, given the pragmatic approach and reliance on widely accepted outcome measures. While certain modifications were made to accommodate the unique needs of our study population, the core elements of the intervention and assessment tools remain applicable for replication. We recommend that future studies consider similar adaptations to ensure the feasibility and relevance of the intervention for diverse patient populations. Quantitative measures provided objective assessments of participants’ functional capacity and symptom severity. Qualitative interviews complemented the quantitative data by offering insights into participants’ perceptions and experiences of the intervention. This qualitative data enriched our interpretation of the quantitative findings, providing context for the study results.

We are aware that this study is not free from limitations. Firstly, the small samples and the absence of comparison severely limit the strength of our results. However, it should be noted that this is the first pilot study providing disease-specific data about a comprehensive rehabilitation intervention in patients with MPM. Moreover, the high number of dropouts after the baseline assessment (*n* = 5) suggested that the therapeutic intervention might only be suitable for some patients with MPM. Thus, a precise evaluation of patients’ characteristics is needed to identify further patients who might receive a comprehensive rehabilitation program to improve symptoms, optimize physical functioning, and enhance the quality of life of people with MPM. 

## 5. Conclusions

Taken together, our findings suggest promising effects of a multidisciplinary approach including educational therapy, counseling, and physical and pulmonary rehabilitation. The tailored nature of the intervention and the use of a home-based program highlight its practical feasibility for real-world implementation. These findings are particularly relevant for healthcare providers seeking to integrate evidence-based rehabilitation strategies into the multidisciplinary care of MPM patients. This pilot study provides valuable insights and a strong foundation for future research, guiding clinical decision-making and the development of comprehensive rehabilitation programs aimed at improving the quality of life for individuals with MPM. Due to the rareness of the disease, further multi-centric studies assessing larger samples and control groups are needed to further characterize the role of rehabilitation interventions in the multidisciplinary tailored management of people with MPM.

## Figures and Tables

**Table 1:** Patients’ baseline characteristics.

Variables	Value
	Patients (*n* = 7)
Age (years)	67 ± 6
Female	1 (14.3%)
Male	6 (85.7%)
Weight (kg)	76.71 ± 12.89
Height (cm)	172 ± 6.71
BMI (kg/m^2^)	26.05 ± 5.04
Smokers	2 (28.6%)
Comorbidities	
*Diabetes Mellitus*	3 (42.9%)
*Myocardial Infarction*	1 (14.3%)
*Chronic Obstructive Pulmonary Disease*	1 (14.3%)
*Arterial Hypertension*	3 (42.9%)
*Benign Prostatic Hypertrophy*	1 (14.3%)
*Dyslipidemia*	2 (28.6%)
Level of physical activity *	
*None*	4 (57.1%)
*Low*	1 (14.3%)
*Medium*	2 (28.6%)
*High*	0 (0.0%)
Histology	
*Epithelioid*	7 (100%)
*Sarcomatoid*	0 (0%)
*Biphasic/mixed*	0 (0%)
Right-side tumor	6 (85.7%)
Left-side tumor	1 (14.3%)
Stage	
*IA*	2 (28.6%)
*IB*	2 (28.6%)
*II*	0 (0%)
*IIIA*	2 (28.6%)
*IIIB*	1 (14.3%)
*IV*	0 (0%)
Grade	
*Low*	2 (28.6%)
*High*	5 (71.4%)
Pleural Surgery	
*Pleurodesis with talc*	4 (57.1%)
*Pleurectomy/decortication*	2 (28.6%)
*No surgery*	1 (14.3%)
Radiotherapy	0 (0.0%)
Chemotherapy	7 (100%)

Continuous variables are expressed as means ± standard deviations, categorical variables are expressed as counts (percentages). Abbreviations: BMI: body mass index, *n*: number. * According to the “World Health Organization Global Recommendations on Physical Activity for Health, Geneva, World Health Organization, 2010”.

**Table 2:** Main results for physical performance parameters.

	T0 (*n* = 7)	T1 (*n* = 7)	T2 (*n* = 7)	T0–T1	T0–T2	MCID	T0–T1	T0–T2
	Mean ± SD	Mean ± SD	Mean ± SD	Mean ± SD	Mean ± SD	Mean	*p* Value	*p* Value
HGS (kg)	31.07 ± 4.51	34.10 ± 9.1	33.5 ± 6.65	−3.03 ± 6.74	−2.43 ± 3.20	5.5	0.375	0.1094
SPPB	11.42 ± 0.79	11 ± 0.82	10.71 ± 1.11	0.43 ± 1.40	0.71 ± 1.60	1	0.625	0.3125
Submaximal Exercise Capacity								
2MWT (m)	132 ± 17.15	149.43 ± 27.61	131.43 ± 23.60	−17.43 ± 23.86	0.57 ± 21.77	5.5	0.1875	0.9531
2MWT (RPE Borg)	2.36 ± 2.14	2.43 ± 1.90	1.86 ± 1.57	−0.07 ± 2.86	0.5 ± 2.7	1	>0.9999	0.67
2MWT (SpO_2_)	96.14 ± 0.9	95.42 ± 43	90.86 ± 3.39	0.71 ± 3.35	5.28 ± 4.07	-	0.7031	0.03 *
30secSTS	9.71 ± 2.29	11.42 ± 2.37	15.57 ± 4.08	−1.71 ± 3.30	−5.86 ± 5.11	2	0.4375	0.046 *

Abbreviations: 2MWT: two-minute-walking test, 30secSTS: 30 s sit-to-stand, HGS: handgrip strength, m: meter, MCID: minimal clinically important difference, *n*: number, SD: standard deviation, SpO_2_: oxygen saturation, SPPB: Short Physical Performance Battery, RPE: rate of perceived exertion. T0: baseline, T0–T1: variation between baseline and the end of treatment, T1: baseline + 1 month, T0–T2: variation between baseline and the end of follow-up, T1: baseline + 6 months, * *p* < 0.05.

**Table 3:** Main results of arterial blood gas test and pulmonary function assessed with spirometry.

	T0 (*n* = 7)	T1 (*n* = 7)	T2 (*n* = 7)	T0–T1	T0–T2
	Mean ± SD	Mean ± SD	Mean ± SD	*p* Value	*p* Value
Arterial Blood Gas Test					
PaO_2_	78 ± 4.64	83.84 ± 6.21	75.68 ± 10.99	0.58	0.99
PaCO_2_	39.79 ± 4.6	41.51 ± 3.15	40.74 ± 4.80	0.16	0.12
pH	7.44 ± 0.04	7.42 ± 0.02	7.40 ± 0.03	0.12	0.12
Pulmonary Function					
FEV1 (L)	2.41 ± 0.81	2.35 ± 0.72	2.14 ± 0.54	0.50	0.16
FVC (L)	2.86 ± 0.92	2.89 ± 0.88	2.66 ± 0.69	0.34	0.22
DLCO (% predicted)	68.17 ± 13.93	65.71 ± 12.43	61.43 ± 12.42	0.91	0.22
PEF	6.61 ± 2.04	7.58 ± 2.09	7.67 ± 1.91	0.09	0.62

Abbreviations: DLCO: Diffusing capacity for carbon monoxide, FEV1: forced expiratory volume in the 1st second, FVC: forced vital capacity, L: liter, *n*: number, PaCO_2_: partial pressure of carbon dioxide, PaO_2_: partial pressure of oxygen, PEF: peak expiratory flow, SD: standard deviation, T0: baseline, T1: baseline + 1 month, T1: baseline + 6 months.

**Table 4:** Main outcomes for the multidimensional assessment of patients with MPM.

	T0 (*n* = 7)	T1 (*n* = 7)	T2 (*n* = 7)	T0–T1	T0–T2	MCID	T0–T1	T0–T2
	Mean ± SD	Mean ± SD	Mean ± SD	Mean ± SD	Mean ± SD	Mean	*p* Value	*p* Value
VAS	20.00 ± 23.27	8.14 ± 10.37	9.29 ± 7.87	11.86 ±18.33	10.71 ± 22.07	10	0.19	0.36
MNA	25.2 ± 2.36	26.57 ± 1.64	25.36 ± 2.72	−1.43 ± 2.32	−0.21 ± 2.46	-	0.89	0.36
EORTC QLQ-C30								
Functional score	25.83 ± 9.30	23.86 ± 7.03	21.57 ± 5.50	2.00 ± 3.37	4.28 ± 4.49	7.5	0.12	0.09
Symptom score	20.67 ± 5.24	18.86 ± 4.78	18.71 ± 3.04	1.57 ± 3.69	1.71 ± 1.98	7.5	0.34	0.12
Global Health score	6.50 ± 1.23	5.85 ± 0.69	6.00 ± 1.00	0.57 ± 1.27	0.43 ± 0.98	7.5	0.50	0.50
PROMIS	131.29 ± 44.35	121.14 ± 35.93	116 ± 32.90	10.14 ± 31.20	15. 29 ± 26.54	4	0.45	0.30
LCSS-meso	29.05 ± 20.25	34.63 ± 20.45	36.75 ± 16.9	−5.57 ± 6.44	−7.70 ± 7.86	-	0.08	0.047 *

Abbreviations: EORTC QLQ-C30: European Organization for Research and Treatment of Cancer Quality of Life Questionnaire, LCSS-meso: Lung Cancer Symptom Scale-Mesothelioma, MCID: minimal clinically important difference, MNA: Mini Nutritional Assessment, *n*: number, SD: standard deviation, *n*: number, PROMIS: Patient-Reported Outcomes Measurement Information System, SD: standard deviation, T0: baseline, T0–T1: variation between baseline and the end of treatment, T1: baseline + 1 month, T0–T2: variation between baseline and the end of follow-up, T1: baseline + 6 months, VAS: Visual Analog Scale. * = significance.

## Data Availability

The data that support the findings of this study are available from the corresponding author, upon reasonable request.

## Supplementary Materials

The following supporting information can be downloaded at: https://www.mdpi.com/article/10.3390/cancers16112023/s1. Supplementary Material S1. Qualitative questionnaire administered to the patient at T2.
