# Supplementary material for: Comprehensive Pulmonary Rehabilitation for Patients with Malignant Pleural Mesothelioma: A Feasibility Pilot Study
Source: Cancers (Basel). 2024 May 26;16(11):2023. doi: 10.3390/cancers16112023 (PMC11171244; doi:10.3390/cancers16112023)
Supplement: Supplementary file 1 [file cancers-16-02023-s001.zip › cancers-2998573-supplementary.odt]

Supplementary Material 1. Qualitative questionnaire administered to the patient at T2.

Perceived effectiveness

- Since you started the rehabilitation program, do you perceive an improvement in your general condition?

- Which symptom do you perceive to benefit the most from the rehabilitation program?

- Which symptom do you perceive to benefit the least from the rehabilitation program?

Barriers encountered during the rehabilitation treatment

- Is the rehabilitation program simple enough to be adapted and performed in your daily routine?

- In your opinion, when is the best time during the day to carry out the rehabilitation program?

- In your opinion, what is the best way to carry out the rehabilitation program during the day (continuously, in several phases, first the exercises and then the breathing machine or vice versa, etc.)?

- When do you think is the most appropriate timing to carry out the program during medical therapy (before treatment, after treatment, etc.)?

- Which points of the rehabilitation program are more difficult to integrate into your daily routine or/and are more difficult to understand and memorize?

- Where do you think it would be best to carry out the rehabilitation program (in the hospital, outpatient clinic, in the community, at home, etc.)?

- In your opinion, which modality would be best to carry out the rehabilitation program (independently, independently and with virtual support, in large groups, in small groups)?

- Would you prefer the rehabilitation group to include people with the same pathology as you or even different conditions?

Impact on quality of life

- Does the rehabilitation program have an impact on the organization of the activities you normally carry out during the day?

- Do you think the program makes your day more difficult and complex?

- Do you think the rehabilitation program has any impact on your medical treatment, quality of life, free time, opportunities for social interaction, interaction with your family, etc? Both positively and negatively.

Psychological and social experiences related to the treatment

- In your opinion, which are the benefits of the rehabilitation program on your pathology?

- What are your hopes regarding the impact of the rehabilitation program on your condition?

- Have you ever experienced fear and frustration regarding a possible ineffectiveness of the rehabilitation program?

- Would you change anything in the rehabilitation program? (Would you do something different or avoid something that might improve the program?)

- Which points of the rehabilitation program were the most interesting?

- Which exercises of the rehabilitation program were the least interesting?

- Do you think this rehabilitation program is underestimated/overestimated or is it appropriate to your condition, your age, your sex, your habits?

- What symptoms do you think would be more appropriate for the rehabilitation program to focus on?

- Does going through the rehabilitation program help you control your emotional states? (For example, if one day you feel blue, does doing the exercises help you lift your spirits?)
